# Autophagy Activation Protects Ocular Surface from Inflammation in a Dry Eye Model In Vitro

**DOI:** 10.3390/ijms21238966

**Published:** 2020-11-26

**Authors:** Zhao Liu, Ding Chen, Xin Chen, Fang Bian, Ning Gao, Jinmiao Li, Stephen C. Pflugfelder, De-Quan Li

**Affiliations:** 1 Ocular Surface Center, Cullen Eye Institute, Department of Ophthalmology, Baylor College of Medicine, Houston, TX 77030, USA; dr.liuzhao@foxmail.com (Z.L.); necoding@126.com (D.C.); chenxineye@gmail.com (X.C.); bftongji@hotmail.com (F.B.); ninggao1225@163.com (N.G.); jinmiao.li@bcm.edu (J.L.); stevenp@bcm.edu (S.C.P.); 2 Department of Ophthalmology, the First Affiliated Hospital of Xi’an Jiaotong University, Xi’an 710061, China; 3 School of Ophthalmology & Optometry, Wenzhou Medical University, Wenzhou 325027, China

**Keywords:** hyperosmolarity, autophagy, inflammation, human corneal epithelial cells, rapamycin

## Abstract

Inflammation is the main pathophysiology of dry eye, characterized by tear film instability and hyperosmolarity. The aim of this study was to investigate the association of inflammation and cellular autophagy using an in vitro dry eye model with primary cultured human corneal epithelial cells (HCECs). Primary HCECs cultured with fresh limbal explants from donors were switched to a hyperosmotic medium (450 mOsM) by adding sodium chloride into the culture medium. We observed the stimulated inflammatory mediators, TNF-α, IL-1β, IL-6 and IL-8, as well as the increased expression of autophagy related genes, Ulk1, Beclin1, Atg5 and LC3B, as evaluated by RT-qPCR and ELISA. The immunofluorescent staining of LC3B and Western blotting revealed the activated autophagosome formation and autophagic flux, as evidenced by the increased LC3B autophagic cells with activated Beclin1, Atg5, Atg7 and LC3B proteins, and the decreased levels of P62 protein in HCECs. Interestingly, the autophagy activation was later at 24 h than inflammation induced at 4 h in HCECs exposed to 450 mOsM. Furthermore, application of rapamycin enhanced autophagy activation also reduced the inflammatory mediators and restored cell viability in HCECs exposed to the hyperosmotic medium. Our findings for the first time demonstrate that the autophagy activation is a late phase response to hyperosmotic stress, and is enhanced by rapamycin, which protects HCECs by suppressing inflammation and promoting cells survival, suggesting a new therapeutic potential to treat dry eye diseases.

## 1. Introduction 

Dry eye is a common disease that affects up to 50% of the population worldwide [1]. It is characterized by ocular surface discomfort, tear film instability, increased osmolarity, as well as inflammation and even damage [2,3,4]. Dry eye often occurs in patients with aging, and those exposed to systemic drugs such as antihistamines, β-blockers, antispasmodics, diuretics and some psychotropic drugs. Sjögren’s syndrome is another common cause of dry eye [2]. In clinical practice, the most frequently employed test of tear film stability is the measurement of the tear film breakup time. In addition, the distribution of micropunctate staining may provide an etiological clue for dry eye [5]. As a result of insufficient production [6] and excessive evaporation [7] of tear, hyperosmolarity has been proven as a contributing factor to the inflammatory reaction of the ocular surface in dry eye patients [8,9] and mouse models [10,11,12]. Our group have also observed the increase of pro-inflammatory cytokines (TNF-α, IL-1β, IL-6 and IL-18), chemokine (IL-8), and matrix metalloproteinases (MMPs) in primary cultured human corneal epithelial cells (HCECs) exposed to hyperosmotic conditions [4,10,13,14,15,16,17]. Therefore, artificial tears as supplementation and anti-inflammatory therapy are the main treatments for most dry eye patients [18]. 

Autophagy is a highly conservative self-degradation pathway [19], which is one of the main intracellular homeostasis control systems for almost all eukaryotic cells [20] by recycling macromolecules, especially in response to stresses such as starvation, hypoxia, infection and inflammation [21]. The phenomenon of autophagy to suppress excessive inflammation was first observed in Atg16/1-deficient mice in 2008 [22]. Autophagy modulates inflammation via effecting the survival, development and homeostasis of inflammatory cells [23,24], and influencing the transcription, processing and secretion of inflammatory mediators [25]. Autophagy is regulated by these cytokines as well [26]. In fact, it is well established that Th1 cytokines, including IFN-γ, TNF-α, IL-1, IL-2, IL-6 and TGF-β, have been shown to have the effects of autophagy inducement, while the classical Th2 cytokines, including IL-4, IL-10 and IL-13, have the effects of inhibition. Autophagy related mechanisms have been known to be involved in the pathogenesis of inflammatory diseases, including chronic obstructive pulmonary diseases [27], infectious diseases [28], pulmonary hypertension [29], cystic fibrosis [30] and Crohn’s disease [31]. These studies on autophagy regulation may prompt the development of potentially therapeutic interventions for inflammatory diseases. 

However, the pathophysiological role of autophagy in regulating inflammation of the dry eye disease has not been well elucidated. Byun and colleagues observed that in Sjögren’s syndrome dry eye, the levels of ATG5 and LC3B II/I were significantly higher in tears and conjunctival epithelium, but decreased after one month of treatment with topical corticosteroid [32]. These findings may suggest that the enhanced autophagy is a protective response to inflammation in dry eye, and it is reduced when the anti-inflammatory therapy works. The phenomenon of autophagy regulating the ocular surface inflammation was also observed in mice [33]. In this study, we explored a protective role of autophagy activation in suppressing inflammation in dry eye condition using an in vitro model with primary HCECs under hyperosmotic stress.

## 2. Results

### 2.1. Inflammatory Mediators Are Largely Stimulatd in Primary HCECs under Hyperosmotic Stress, an In Vitro Dry Eye Model

**Figure 1:**
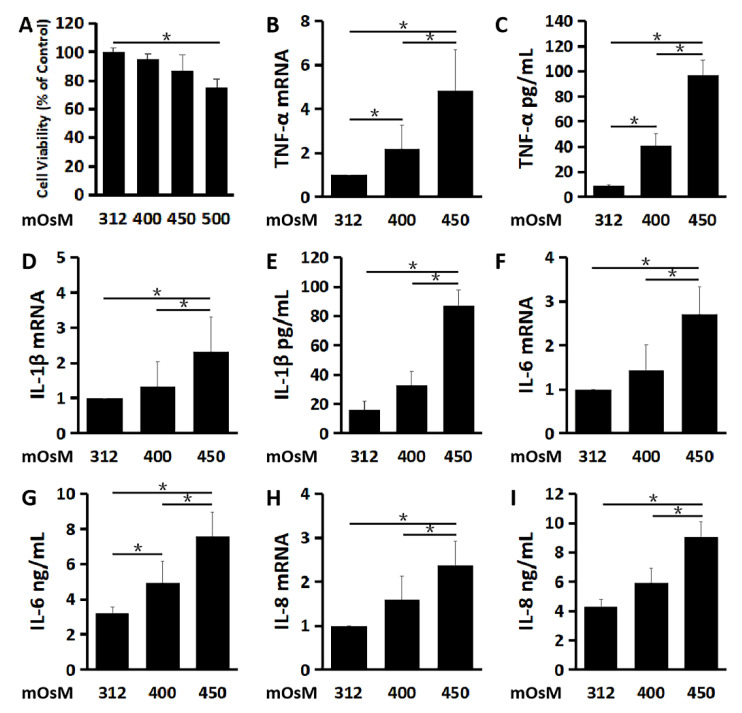
Hyperosmolarity stimulates inflammatory mediators in primary cultured human corneal epithelial cells (HCECs). (**A**) Cell viability of HCECs exposed to different osmolarities analyzed by MTT assay. The pro-inflammatory cytokines, TNF-α (**B**,**C**), IL-1β (**D**,**E**) and IL-6 (**F**,**G**), as well as chemokine IL-8 (**H**,**I**) significantly increased at mRNA and protein levels in HCECs exposed to a hyperosmolar medium at 400 and 450 mOsM for 4 and 24 h, respectively, compared with isosmolar control at 312 mOsM as evaluated by RT-qPCR and ELISA. Data are presented as mean ± SD of 3 independent experiments. * *p* < 0.05, compared between two groups.

Primary HCECs cultured in the isosmolar medium (312 mOsM), were switched to the hyperosmolar medium (400, 450 or 500 mOsM) with the addition of 44, 69 or 94 mM of NaCl, respectively. As evaluated by MTT cell viability assay (Figure 1A), HCECs survived well in the medium at 400 and 450 mOsM while some cells may be damaged with lower viability rates in 500 mOsM medium (*p* < 0.05, compared with 312 mOsM). Therefore, 500 mOsM was excluded for the subsequent study. Both the mRNA expression and protein production of pro-inflammatory cytokines TNF-α (Figure 1B,C), IL-1β (Figure 1D,E) and IL-6 (Figure 1F,G), as well as chemokine IL-8 (Figure 1H,I) were greatly increased with the increasing osmolarity. 

TNF-α mRNA expression by HCECs was sharply stimulated to 2.17 ± 1.11 and 4.82 ± 1.89 fold respectively (*n* = 3, Figure 1B) as the osmolarity increased to 400 and 450 mOsM. ELISA results also showed that TNF-α protein concentrations in the culture supernatant were also significantly increased from 8.71 ± 1.29 pg/mL in the isosmolar medium to 40.67 ± 9.87 and 96.43 ± 12.64 pg/mL in the hyperosmolar medium at 400 and 450 mOsM (*n* = 3, Figure 1C), respectively. 

IL-1β mRNA expression increased to 2.32 ± 0.98 fold (*n* = 3, Figure 1D), accompanied by the elevated IL-1β protein concentrations to 86.96 ± 10.79 pg/mL, (*n* = 3, Figure 1E), in the supernatant of the cultures at 450 mOsM, compared with 16.05 ± 6.21 pg/mL in the isosmolar medium as controls. 

IL-6 mRNA expression increased to 2.71 ± 0.63 fold when exposed to the 450 mOsM medium (*n* = 3, Figure 1F). Meanwhile, IL-6 protein was measured at 4.93±1.23 and 7.57 ± 1.38 ng/mL, respectively (*n* = 3, Figure 1G), in the hyperosmolar medium supernatants at 400 and 450 mOsM, significantly higher than that in isosmolar controls (3.18 ± 0.37 ng/mL). 

The mRNA expression of chemokine IL-8 was raised to 2.37 ± 0.57 fold (*n* = 3, Figure 1H) in the cells at 450 mOsM, and protein levels increased to 9.04 ± 1.07 ng/mL (*n* = 3, Figure 1I), when compared with 4.30 ± 0.49 ng/mL in the isosmolar controls. 

### 2.2. Hyperosmolarity Induces Autophagosome Formation and Promotes Autophagic Flux in Primary HCECs 

**Figure 2:**
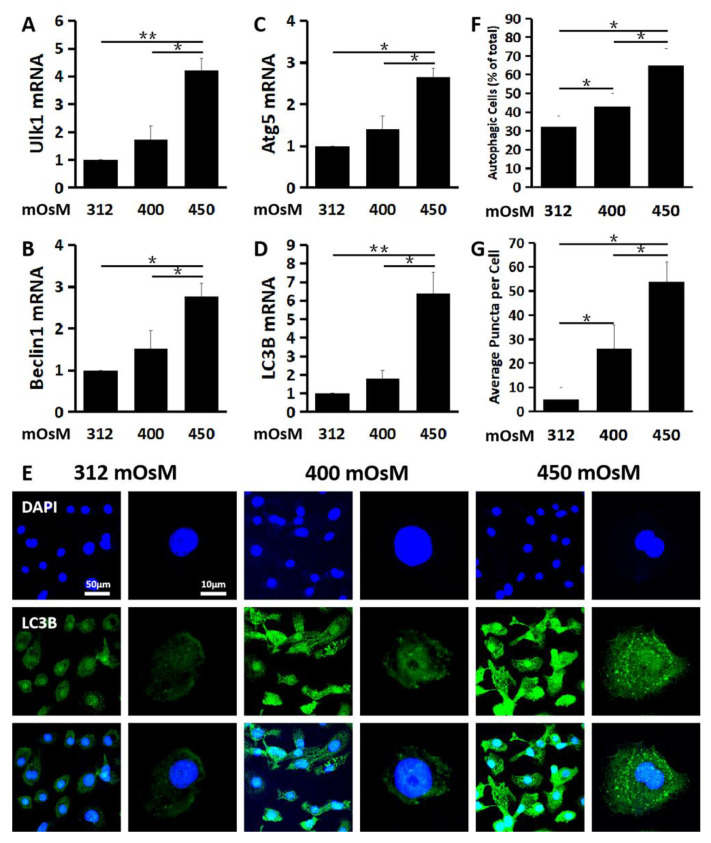
Hyperosmolarity induced autophagosome formation in primary HCECs. The mRNA expression of Ulk1 (**A**), Beclin1 (**B**), Atg5 (**C**) and LC3B (**D**) was significantly induced in HCECs exposed to hyperosmolar medium at 450 mOsM for 24 h, as evaluated by RT-qPCR. (**E**) Representative immunofluorescent images showing the LC3B positive cells with punctate staining in HCECs at various osmotic conditions. (**F**) Percentage of LC3B positive autophagic cells in HCECs at different osmolarities, evaluated by randomly selected fields with at least 100 cells per sample. (**G**) Average puncta per autophagic cell. The data are presented as mean ± SD from three independent experiments. * *p* < 0.05, ** *p* < 0.01, compared between two groups.

Autophagy begins with activation of Ulk1 in the initiation phase, which stimulates Beclin1 in the nucleation phase to regulate and generate formation of the nascent autophagosome in the elongation phase whereby cytosolic components are enclosed into an isolation membrane. We found that the expression of Ulk1 (Figure 2A) in the initiation phase, Beclin1 (Figure 2B) in the nucleation phase and Atg5 (Figure 2C) and LC3B (Figure 2D) in the elongation phase were all significantly activated in HCECs exposed to hyperosmolar medium at 450 mOsM, as evaluated by RT-qPCR. Ulk1 mRNA expression was stimulated to 1.74 ± 0.49 and 4.21 ± 0.43 fold, Beclin1 mRNA was stimulated to 1.51 ± 0.43 and 2.76 ± 0.32 fold and Atg5 mRNA increased to 1.41 ± 0.30 and 2.65 ± 0.22 fold, respectively, when exposed to 400 and 450 mOsM medium (*n* = 3, Figure 2A–C). 

LC3B is a marker commonly associated with autophagy activation with autophagosome formation. LC3B mRNA levels increased moderately to 1.82 ± 0.43 but dramatically to 6.39 ± 1.17 fold, respectively, in response to hyperosmolar medium with 400 and 450mOsM (Figure 2D). The immunofluorescent staining showed that the LC3B positive puncta cells greatly increased in HCECs exposed to 450 mOsM medium when compared with that in the 400 mOsM and isosmolar medium (312 mOsM) (*n* = 3, Figure 2E,F). Besides, the average LC3B puncta per cell were also much more abundant in HCECs exposed to 450 mOsM medium (*n* = 3, Figure 2G). 

### 2.3. Autophagy Activation Is a Late Response to Hyperosmolarity after Inflammation in HCECs 

**Figure 3:**
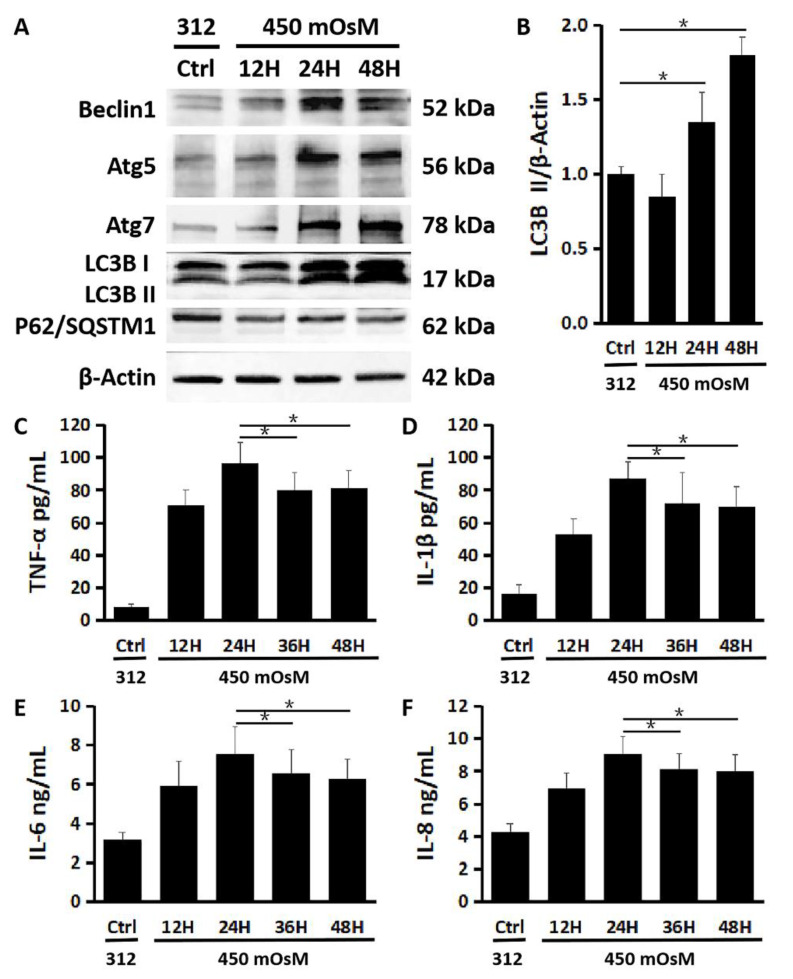
Time-dependent effect of hyperosmotic stress on autophagy activation and inflammation in primary HCECs. (**A**) Western blot showed that the protein production of Beclin1, Atg5 and Atg7, as well as LC3B turnover increased while production of p62/SQSTM1 decreased significantly in 24–48 h after HCECs were exposed to 450 mOsM medium, with β-Actin serving as internal control. (**B**) Quantitative ratio of LC3B II/β-Actin showing increased LC3B turnover in 24–48 h after HCECs were exposed to 450 mOsM. (**C**–**F**) ELISA analyses. The pro-inflammatory cytokines, TNF-α (**C**), IL-1β (**D**) and IL-6 (**E**), as well as chemokine IL-8 (**F**) was suppressed after 24 h in HCECs exposed to 450 mOsM. Data are mean ± SD of three independent experiments. * *p* < 0.05, compared within groups.

We observed that the production levels of autophagy activation related proteins Beclin1, Atg5, Atg7 and LC3B were increased mainly within 24–48 h after HCECs were exposed to 450 mOsM, as evaluated by Western blot (Figure 3 A). The turnover of LC3B I protein to the activated form LC3B II also increased significantly in 24–48 h after longer hyperosmolarity exposure periods (Figure 3A,B, *p* < 0.05, control vs. 24 or 48 h, *n* = 3). Interestingly, the production of p62 protein, also known as SQSTM1, decreased significantly in 24–48 h, indicating the autophagic flux enhanced by hyperosmolarity (Figure 3A). 

Furthermore, a moderate drawdown of pro-inflammatory cytokines TNF-α, IL-1β and IL-6, as well as chemokine IL-8, was detected by ELISA after 24 h exposure (24 h vs. 36 or 48 h, *p* < 0.05; *n* = 3, Figure 3C–F). 

### 2.4. The Enhanced Autophagy Activation by Rapamycin Suppresses Inflammation and Restores the Cell Viability Damaged by Hyperosmolarity 

**Figure 4:**
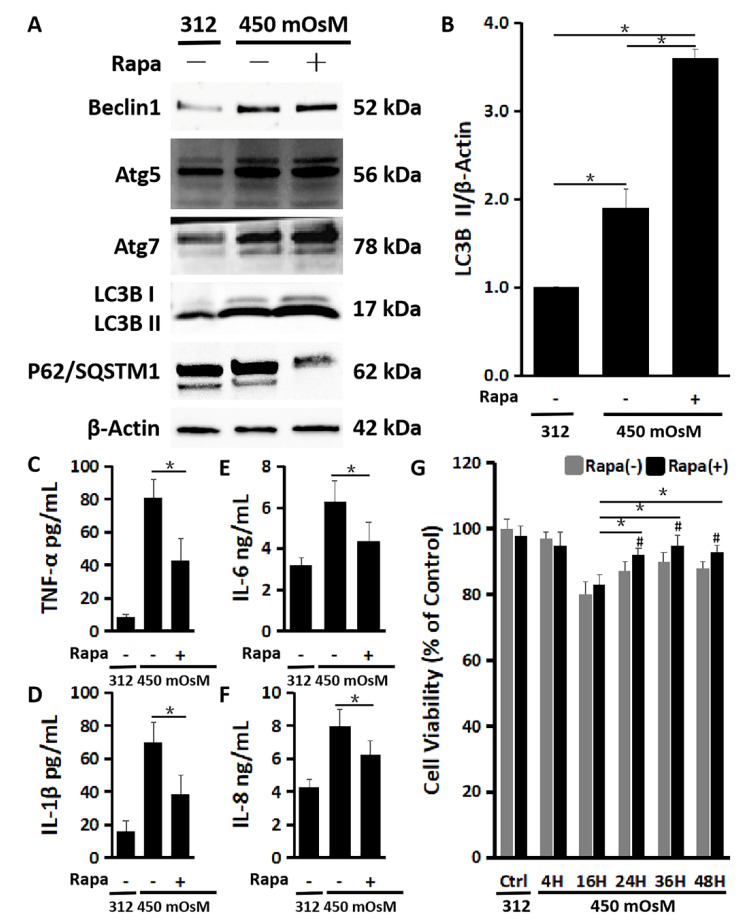
Rapamycin enhanced autophagy activation and protected the cells from inflammation and reduced viability in primary HCECs exposed to hyperosmolarity. (**A**) Western blot showed that 200 nM of rapamycin increased production of Beclin1, Atg5, Atg7 and LC3B while it decreased p62 production in HCECs exposed to 450 mOsM for 2 h, with β-Actin as internal control. (**B**) Quantitative analysis of relative turnover levels of LC3B II/β-Actin. (**C**–**F**) ELISA results showed that the release of TNF-α (**C**), IL-1β (**D**), IL-6 (**E**) and IL-8 (**F**) were reduced by 200 nM of rapamycin in HCECs exposed to 450 mOsM medium for 2 h. (**G**) MTT assay showed that the cell viability was largely rescued by rapamycin after 24 h in HCECs exposed to 450 mOsM medium when compared with cell viability in normal medium (312 mOsM) at 24 h as controls. Data are presented as mean ± SD of three independent experiments. * *p* < 0.05, compared between two groups. ^#^ *p* < 0.05, compared within the same time point.

To confirm the protective role of autophagy activation, HCECs were switched to 450 mOsM medium without or with addition of an autophagy inducer rapamycin. As shown in Figure 4, the autophagy activation was further enhanced by rapamycin (200 nM) in HCECs exposed to 450 mOsM medium, evidenced by the highest production of Beclin1, Atg5, Atg7 and LC3B with the lowest level of P62/SQSTM1 protein when compared with the cells without rapamycin addition (Figure 4A). The LC3B II turnover from LC3B I also sharply increased with rapamycin co-incubation (Figure 4B). 

Meanwhile, the release of inflammatory cytokines was suppressed by rapamycin, evidenced by much lower levels of TNF-α (43.17 ± 12.64 pg/mL), IL-1β (40.11 ± 11.06 pg/mL), IL-6 (4.38 ± 0.84 ng/mL) and IL-8 (6.13 ± 0.79 ng/mL) protein levels in the medium supernatants (Figure 4C–F). Interestingly, rapamycin significantly rescued the cell viability reduced by hyperosmolarity, especially after 2 h (16 h (83.1 ± 3.2%) Rapa (+) vs. 24 h (91.8 ± 2.1%), 36 h (95.0 ± 2.8%) or 48 h (93.2 ± 1.8%) Rapa (+), *p* < 0.05; Rapa (−) vs. Rapa (+) at 24 h (87.9 ± 3.0% vs. 91.8 ± 2.1%), 36 h (90.3 ± 2.9% vs. 95.0 ± 2.8%), or 48 h (88.2 ± 2.0% vs. 93.2 ± 1.8%), *p* < 0.05; Figure 4G). 

## 3. Discussion 

Hyperosmolarity and inflammation are the key pathogenesis in dry eye disease, as acknowledged by TFOS DEWS II [2]. As a result of inadequate production [6] and/or evaporation of tear [7], hyperosmolarity has been proven to be an important element, which generates the inflammation [8,9,10,11,12]. Increasing evidence demonstrates that autophagy influences the pathogenesis of inflammatory diseases [23]. In this study, we found the experimental evidence that autophagy activation is a late response to hyperosmotic stress in primary cultured HCECs. This would help to better understand the role of autophagy regarding the modulation of ocular surface inflammation and cell damage induced by hyperosmolarity. The protective role of autophagy inducer rapamycin would provide clues to the new direction of treating dry eye disease. 

Previous studies, including ours, have confirmed that hyperosmolarity can stimulate an inflammatory reaction via different pro-inflammatory cytokines and chemokine [8,34,35,36], of which an increase has been determined in the primary cultured HCECs in vitro and the murine dry eye model in vivo, as well as the dry eye patients’ tear [13,14]. We herein confirmed the previous findings that HCECs exposed to hyperosmolarity secreted inflammatory mediators such as TNF-α, IL-1β, IL-6 and IL-8. 

Our study further observed the increase of mRNA expression levels of Ulk1 in initiation phase, Beclin1 in nucleation phase, as well as Atg5 and LC3B in elongation phase during autophagosome formation, which reveal the autophagy activation, an instinctive response to stress [37]. The process of autophagy can be triggered in cells under stress conditions such as high levels of inflammatory cytokines stimulated by hyperosmolarity. Moreover, we found that the LC3B autophagic HCECs and average puncta per cell were more abundant as osmolarity increased, which means that autophagosomes formed. 

The formation of autophagosome, detected by fluorescence of LC3B, not only prompts autophagic activation, but also prompts autophagy dysregulation, such as slowed lysosomal degradation or inefficient fusion due to a lack of lysosomal fusion. Considering this, it is essential to monitor the autophagy flux, which is the degradative process that the autophagosome fuses with the lysosome and subsequently breaks down. The autophagy marker LC3B and substrates such as P62/SQSTM1 protein are the common autophagy reporters [38]. P62/SQSTM1 acts as a connection between LC3B and ubiquitinated substrates and is efficiently degraded by autophagy. Consequently, the P62 level in combination with LC3B II turnover can be measured to monitor autophagic flux [39]. In this study, we observed the activation of autophagy in HCECs exposed to hyperosmolarity occurred after 24 h, evidenced by the higher turnover of LC3B I to form LC3B II as well as a decreased P62 level. 

The crosstalk between inflammation and autophagy has recently become an interesting topic, although the mechanism is not fully elucidated [23,24,40,41]. Some studies have shown that autophagy can well balance inflammatory responses to maintain the homeostasis [40,41]. It is suggested that the interaction between inflammation and autophagy may make sense [33,42]. The crosstalk between inflammation and autophagy is complicated. Some forms of autophagy suppress inflammation, which causes collateral cell and tissue damage [43]. Contrarily, a properly ascended, concentrated and transient inflammation promotes cell and tissue repair and regeneration [23]. Ma and colleagues [33] recently showed that autophagy could regulate ocular surface inflammation in female C57BL/6 mice housed in a controlled environment by subcutaneous injection of scopolamine. 

In this study, we interestingly found that hyperosmolarity triggered autophagy activation later than inflammation in primary HCECs. Different from the inflammation factors activated at 4 h, autophagy is a late response to hyperosmotic stress in HCECs at 24 h. After autophagosome formation in HCECs exposed to hyperosmolarity, evidenced by the increases of Beclin1, Atg5, Atg7 and LC3B, as well as the decrease of P62, a few upward blips in cell viability were observed, accompanied by a moderate drawdown of TNF-α, IL-1β, IL-6 as well as IL-8. Autophagy modulates inflammation through influencing the inflammatory cells survival conditions and altering secretion of inflammatory cytokines [33,44], by which autophagy is simultaneously modulated [24]. Thus, we assessed how modulators of autophagy can relieve ocular surface inflammation linked with dry eye disease. 

To confirm the protective role of autophagy activation, we further performed the experiments using an autophagy inducer rapamycin. We observed that rapamycin did not show its protective effect on HCECs in hyperosmolarity within 4–16 h. It is supposed that the activation of autophagy is still a late response by HCECs with rapamycin. The autophagy activation was significantly enhanced after 24 h, when rapamycin may accelerate its effect, and then suppress the secretion of inflammatory cytokines and chemokine. The cell viability reduced by hyperosmolarity was also restored by rapamycin. The findings established a link between the autophagy activation by rapamycin and the restoration of cell homeostasis. 

Further investigation is needed to explore the potential regulation mechanism of autophagy in the pathogenesis of dry eye at different stages. Thorough illustration of the mechanism of autophagy and its interaction with other pathological events, such as inflammatory response and oxidative stress [15,34,45,46], will help us to better understand the pathophysiology of dry eye and other ocular surface diseases. Thus, treatments targeting autophagy and inflammation together might find clinical applications. In this connection, autophagy inducers and inhibitors are needed to further identify the potential therapeutic role of rapamycin in treating the dry eye condition. 

## 4. Materials and Methods 

### 4.1. Cultures of Primary HCECs and Dry Eye Model In Vitro 

Fresh human corneal tissues not suitable for transplant were provided by the Lions Eye Bank of Texas (Houston, TX, USA). The corneal tissues were obtained from donors within 72 h after death (age of donors ranged from 18–65 years). Primary HCECs were cultured from donors’ limbus explants in a supplemented hormonal epidermal medium (SHEM) containing 5% FBS as our previous publications [47]. Fourteen to eighteen days later, confluent cultures of HCECs were switched to a serum-free SHEM without 5% FBS for 24 h. The culture medium osmolarity was monitored by a vapor pressure osmometer in the Body Fluids Clinical Chemistry Laboratory of the Houston Methodist Hospital–Texas Medical Center (Houston, TX, USA) [4]. The addition of 44, 69 and 94 mM of sodium chloride (NaCl) can achieve hyperosmolarity (400, 450 and 500 mOsM) from the isosmolar (312 mOsM) medium. The HCECs co-incubated for 4 h were used for total RNA extraction and lysed in buffer RLT plus from the Qiagen RNeasy Plus Mini kit (#74134, QIAGEN, Germantown, MD, USA). The HCECs co-incubated for 12 h, 24 h or 48 h were used for immunostaining or lysed in RIPA buffer (Sigma-Aldrich, St. Louis, MO, USA) for Western blot analysis. 200 nM of Rapamycin (an autophagy activator, Sigma-Aldrich, St. Louis, MO, USA) was used for autophagy regulation. The supernatant of the conditioned medium was prepared for enzyme-linked immunosorbent assay (ELISA). 

### 4.2. Real-Time Quantitative Polymerase Chain Reaction (RT-qPCR) 

Total RNA extraction was conducted with the RNeasy Plus Mini Kit (QIAGEN, Germantown, MD, USA), quantified with a NanoDrop^TM^ 2000 spectrophotometer (Thermo Fisher Scientific, Waltham, MA, USA). Then, the first strand cDNA was synthesized by reverse transcription using 2.0 μg of the total RNA extraction and Ready-To-Go You-Prime First-Strand Beads (GE Healthcare, Piscataway, NJ, USA). As per our previous described methods [48], RT-qPCR was carried out in a StepOnePlus™ Real-Time PCR System (Applied Biosystems, Foster City, CA, USA) with 10.0 μL reaction volume containing 4.5 μL of cDNA, 0.5 μL gene expression assay and 5.0 μl TaqMan™ Fast Universal PCR Master Mix (Applied Biosystems, Carlsbad, CA, USA). Taqman^®^ Gene Expression Assays (Applied Biosystems, Carlsbad, CA, USA) were: TNF-α (Hs00174128_m1), IL-1β (Hs01555413_m1), IL-6 (Hs00174131_m1), IL-8 (Hs00174103_m1), as well as GAPDH (Hs99999905_m1) as an internal control. The results were analyzed by the comparative threshold cycle method and normalized by GAPDH as an internal control.

### 4.3. Enzyme-Linked Immunosorbent Assay (ELISA) 

Double-sandwich ELISA for human TNF-α, IL-1β, IL-6 and IL-8 (BioLegend, San Diego, CA, USA) was conducted to determine the protein concentration of these mediators in the conditioned medium with different treatments [14]. Absorbance was read at 450 nm with 570 nm as a reference wavelength by Infinite^®^ M200 PRO multimode microplate readers (Tecan US, Morrisville, NC, USA). 

### 4.4. Western Blot Analysis 

As described in our previous publication [4], equal amounts of protein, measured by a Micro BCA Protein Assay Kit (Pierce Biotechnology, Rockford, IL, USA), were mixed with 6 × SDS reducing sample buffer and boiled for 5 min before loading. The proteins (50 μg/lane) were separated on a 4–15% Mini-PROTEAN^®^ TGX Stain-Free™ Protein Gel (Bio-Rad Laboratories, Hercules, CA, USA) and transferred electrophoretically to 0.2 μm PVDF membranes (Bio-Rad Laboratories, Hercules, CA, USA). The membranes were blocked with 5% nonfat milk in TTBS for 1 h at room temperature and incubated with primary antibodies against human Beclin1, ATG5, ATG7, LC3B, P62/SQSTM1 (Novus Biologicals, Littleton, CO, USA), or β-Actin (Boster Biological Technology, Pleasanton, CA, USA) overnight at 4 °C. After five washes with TTBS for 5 min each, the membranes were incubated with HRP conjugated Goat anti-Rabbit IgG (H+L) Secondary Antibody (1:10,000, Thermo Fisher Scientific, Rockford, IL, USA) or Goat anti-Mouse IgG (H+L) Cross-Adsorbed Secondary Antibody (1:10,000, Thermo Fisher Scientific, Rockford, IL, USA) for 1 h at room temperature. The signals were detected with a Clarity™ Western ECL Substrate (Bio-Rad Laboratories, Hercules, CA, USA), and the images were obtained by a ChemiDoc™ MP Imaging System (Bio-Rad Laboratories, Hercules, CA, USA). 

### 4.5. Immunofluorescent Staining 

Primary HCECs, cultured on 8-chamber slides, were fixed with methanol at –20 °C for 5 min. Indirect immunofluorescent staining was conducted as our previously described [49]. Primary rabbit polyclonal antibody against human LC3B (Novus Biologicals, Littleton, CO, USA) was used. Alexa Fluor^®^ 488-conjugated AffiniPure Goat Anti-Rabbit IgG (H+L) secondary antibody (Jackson ImmunoResearch Laboratories, West Grove, PA, USA) was applied, and 4′,6-diamidino-2-phenylindole (DAPI, Invitrogen, Eugene, OR, USA) was used for nuclear counterstaining. As a negative control, secondary antibody was applied alone and compared to isotype goat IgG. The staining was then photographed with Nikon A1 Confocal Laser Microscope System (Nikon Instruments, Melville, NY, USA) and processed with Image J software (National Institutes of Health, Bethesda, MD, USA). 

### 4.6. MTT Assay 

Primary HCECs were seeded at 2 × 10^4^ cells/ml onto 96-well plates with 200 μL/well of SHEM overnight, and treated for 24 h or different time periods (4, 16, 24, 36 and 48 h) in isosmolar (312 mOsM) or hyperosmolar medium (450 mOsM), with 1 h prior co-incubation with rapamycin (Sigma-Aldrich, St. Louis, MO, USA) or not. The proliferative activity of the cells was quantitatively measured by a Cell Growth Determination Kit, MTT based (Sigma-Aldrich, St. Louis, MO, USA). The optical density (OD) of absorbance at 570 nm was read by Infinite^®^ M200 PRO multimode microplate readers (Tecan US, Morrisville, NC, USA) [50]. 

### 4.7. Statistical Analysis 

Student’s *t*-test was used to compare two groups. One-way ANOVA test was used to compare three or more groups, followed by Dunnett’s post-hoc test to compare within groups. *p* < 0.05 was considered statistically significant. 

## 5. Conclusions

Our findings for the first time demonstrate that the autophagy activation is a late response to hyperosmotic stress after triggering inflammation in HCECs, which protects HCECs by reducing the inflammatory mediators and promote sell survival. These protective effects are further strengthened when the autophagy activation is enhanced by rapamycin in HCECs exposed to hyperosmolarity, an in vitro dry eye model, suggesting a new therapeutic potential to treat dry eye diseases. Further investigations are needed to explore the mechanisms of autophagy regulation underlying in the pathogenesis of dry eye diseases. 

## Abbreviations

HCECs	human corneal epithelial cells
IL	interleukin
MMPs	matrix metalloproteinases
RAPA	rapamycin
SHEM	supplemented hormonal epidermal medium
NaCl	sodium chloride
